# Errata

**Published:** 1783

**Authors:** 


					p. 104,
/. I4> for Dificrtatio read. Delmeatio j

				

